# A Virtual Object-Location Task for Children: Gender and Videogame Experience Influence Navigation; Age Impacts Memory and Completion Time

**DOI:** 10.3389/fpsyg.2018.00451

**Published:** 2018-04-04

**Authors:** David Rodriguez-Andres, Magdalena Mendez-Lopez, M.-Carmen Juan, Elena Perez-Hernandez

**Affiliations:** ^1^Instituto Universitario de Automática e Informática Industrial, Universitat Politècnica de València, Valencia, Spain; ^2^IIS Aragón, Departamento de Psicología y Sociología, Universidad de Zaragoza, Zaragoza, Spain; ^3^Departamento de Psicología Evolutiva y de la Educación, Universidad Autónoma de Madrid, Madrid, Spain

**Keywords:** virtual environment, behavior, emotion, short-term memory, visuospatial skill, children

## Abstract

The use of virtual reality-based tasks for studying memory has increased considerably. Most of the studies that have looked at child population factors that influence performance on such tasks have been focused on cognitive variables. However, little attention has been paid to the impact of non-cognitive skills. In the present paper, we tested 52 typically-developing children aged 5–12 years in a virtual object-location task. The task assessed their spatial short-term memory for the location of three objects in a virtual city. The virtual task environment was presented using a 3D application consisting of a 120″ stereoscopic screen and a gamepad interface. Measures of learning and displacement indicators in the virtual environment, 3D perception, satisfaction, and usability were obtained. We assessed the children’s videogame experience, their visuospatial span, their ability to build blocks, and emotional and behavioral outcomes. The results indicate that learning improved with age. Significant effects on the speed of navigation were found favoring boys and those more experienced with videogames. Visuospatial skills correlated mainly with ability to recall object positions, but the correlation was weak. Longer paths were related with higher scores of withdrawal behavior, attention problems, and a lower visuospatial span. Aggressiveness and experience with the device used for interaction were related with faster navigation. However, the correlations indicated only weak associations among these variables.

## Introduction

Loomis et al. (1999) reviewed the potential of immersive virtual environment (VE) technology for experimental psychology. They described its value as a tool in research on spatial cognition. They highlighted its advantages in terms of methodological issues, that are difficult to achieve in practice without this type of technological support (e.g., facilitating the control of the delivered stimuli, manipulating variables, recording measurements and allowing exposure to complex and natural-appearing environments). VEs have also become quite popular for their contributions to neuropsychological assessment. Measures of performance (e.g., correct responses and completion time) derived from tasks using VEs have shown moderate sensitivity in detecting cognitive impairments in clinical populations, especially in the assessment of visuospatial and memory skills (see the review of Negut et al., 2016). Some VEs have been used to study children’s performance, reporting differences between typically-developing children and children with developmental issues (Bioulac et al., 2012; Courbois et al., 2013; Kalyvioti and Mikropoulos, 2013; Broadbent et al., 2015; Farran et al., 2015). Therefore, virtual reality-based tasks currently play an important role in the field of child psychological assessment as an adjunct to standardized classical tests.

The study of human spatial cognition using VEs became quite popular by emulating virtual tasks based on animal mazes (e.g., Astur et al., 2004; Cánovas et al., 2008). Other virtual tasks simulated familiar places for humans (e.g., Maïano et al., 2011; Purser et al., 2012; Burles et al., 2014). The VEs can be viewed on a computer screen (e.g., Astur et al., 2004; Merrill et al., 2016) or other virtual reality platforms, such as head-mounted displays (HMDs), which can provide a full 360° view (Siemerkus et al., 2012). In a typical spatial task, the person controls their movements in the virtual space to memorize places, objects, or routes using a joystick (e.g., Astur et al., 2004; Siemerkus et al., 2012; Walkowiak et al., 2015) or a keyboard (e.g., Purser et al., 2012; Merrill et al., 2016).

Virtual environments used for researching spatial navigation abilities in children have been very similar to those used for adult research (e.g., Hamilton et al., 2003; León et al., 2014; Broadbent et al., 2015; Nys et al., 2015). Children have been asked to navigated the VE and then were tested on their ability to retrace routes or to memorize places or objects. The results found can be extrapolated to results obtained within real environments (e.g., Schmelter et al., 2009). Also, these virtual tasks have been used to draw conclusions about difficulties in orientation in children with developmental disorders (e.g., Hamilton et al., 2003; Courbois et al., 2013; Fornasari et al., 2013; Broadbent et al., 2015).

Most of the studies looking at factors influencing children’s performance in spatial tasks have been focused on cognitive variables such as visuospatial abilities (e.g., Nys et al., 2015), memory (e.g., Purser et al., 2012; Nys et al., 2015), executive functions (e.g., Purser et al., 2012), or navigational strategies (e.g., Bullens et al., 2010; León et al., 2014). The impact of children’s non-cognitive skills on spatial task performance has been less studied. Van den Brink and Janzen (2013) considered the effects of self-care skills measured using the Vineland Screener. The authors found that there was a significant relation between adaptive functioning and the performance of 2 to 3-year-olds on a VR spatial task used for the assessment of orientation skills. They suggested that independence in everyday activities presented by some of the children was critical in improving their spatial knowledge because of a greater number of opportunities for exploring their spatial surroundings. Also, exploratory behavior was related to emotional factors in a study that tested children with autism (Fornasari et al., 2013) who were less active during free exploration of a virtual town. Children suffering from anxiety disorder also performed more poorly than control participants in a virtual Morris water maze (Mueller et al., 2009). They showed thigmotaxis behavior (i.e., the adaptive tendency to avoid exploring the central zone of a novel place) at the beginning of the test and higher numbers of heading errors and unsuccessful trials. Psychometrical measures of anxiety, but not depression, were related to the number of heading errors.

To our knowledge, there are no published studies about relationships between emotional factors and spatial performance in VEs in healthy children. We suggest that affective components and adaptive behavior could influence the performance of typically-developing children in a spatial task involving exploration of a VE. Previous studies performed in adults found that thigmotaxis behavior was positively correlated with affective components (Kallai et al., 2007). These results were obtained after controlling for gender differences in the levels of fear (i.e., women scored higher than men). Also, neuroticism and psychoticism traits had a negative impact on spatial performance (Burles et al., 2014; Walkowiak et al., 2015). These studies yielded conclusions for young adults, but little is known about the relationships between these psychological variables and performance in the child population.

In the present study, we aimed to determine if the performance of typically-developing children in an emotionally neutral virtual-based spatial task is related to their behavioral and emotional outcomes. To do this, we used a basic short-term memory test in which children were to learn the spatial locations of objects (i.e., the learning phase) and later were asked about the correct position of one of these objects (i.e., the testing phase). The VE of this virtual object-location (VOL) task consisted of a city square. To provide visual guidance, the square was surrounded by distal cues and proximal cues. The objects were associated to a place holder and located in the central area of the VE. The VE was presented using a 3D application consisting of a 120″ stereoscopic screen. The children could actively explore details required for orientation by traveling across the interaction area taking a first-person perspective. We chose a gamepad as the device for interaction because it has been preferred by children due to its playability (Rodríguez-Andrés et al., 2016). We tried to reduce the potential influence of individual differences in the experience with videogames and gamepads. For this reason, the participants were trained with the gamepad and performed a tutored trial of the task.

The VOL task was similar to the task used in Rodríguez-Andrés et al. (2016) in terms of the visual and procedural aspects; however, in the present study, we aimed to obtain information about how children’s exploratory behaviors were. The present task assessed not only the ability of the children to recall the place of the objects, but also their way of exploring the interaction area of the VE. The aims of the study by Rodríguez-Andrés et al. (2016) were mainly to present the task, to validate the task for the assessment of spatial short-term memory, and to examine the influence of the type of interaction used on the ability to recall the place of the objects, and the perceived usability and satisfaction of the children with the task.

We obtained objective performance measures of the participating children on the VOL task (i.e., learning and displacement indicators). We assessed their perception about the task (3D perception, satisfaction, and usability) and previous videogame experience. We also considered participant individual differences in the performance of small-scale visuospatial tasks (building blocks and visuospatial span), emotional outcomes (i.e., anxiety, depression, and aggressiveness), and behavioral outcomes (i.e., hyperactivity, withdrawal, and attention problems), which were obtained with a psychometric rating scale. We also considered the age and gender of the participants. The research questions are: (1) Does age, gender, or previous videogame experience of the children affect their performance on the VOL task?; and (2) Are there any significant relationships among performance of the VOL task and the user’s variables (i.e., videogame experience, ratings about the experience with the VOL task, visuospatial skills, emotional and behavioral outcomes)?

We hypothesized that age would affect performance in the VOL task. We studied a wide age range, as the values of the learning indicators would be lower in children younger than 6 years. We did not expect to find an effect of gender on VOL task performance because of its low level of difficulty. The task involved remembering the locations of three objects that were shown sequentially with several proximal visual cues aiding orientation. Both boys and girls might use specific orientation strategies to solve the task. We did not expect to find an effect of videogame experience on task execution because the participants were trained before being tested. Finally, a higher ability for recalling objects in the VE would be linked with higher visuospatial skills on small-scale tests. The displacements made across the interaction area of the VE during performance of the task would be related to emotional and behavioral outcomes. Specifically, higher scores on anxiety would be related to a higher tendency to explore the boundaries of the VE.

## Materials and Methods

### Participants

The participants were 52 right-handed, typically-developing children from 5 to 12 years old (22 girls and 30 boys; M_age_ ± SD = 8.06 ± 1.60). They were divided into three age groups: preschool (5–6 year olds; 5 boys and 4 girls); the first cycle of primary school (7–9 year olds; 18 boys and 13 girls); and the second cycle of primary school (10–12 year olds; 7 boys and 5 girls). They were recruited from a summer school. This final sample was selected after applying the inclusion criteria to a larger sample composed of 66 children. None of the participating children had visual or hearing impairments or had had a breech birth, required neonatal resuscitation, had a body temperature higher than 40° in the first 5 years of life, had suffered a brain injury, had any impairment in motor performance, or were treated with a medication that could potentially impair their cognitive functioning. A questionnaire was completed by their parents concerning their development and medical history. The parents also completed the Movement ABC-2 Checklist (Henderson et al., 2012) to discard any motivational or emotional difficulty related to motor tasks. We used the Lang-Stereo-Test (Lang, 1983) to check that the children could perceive 3D properly. All parents gave written informed consent before their children’s participation. The study was conducted in accordance with the European Directive 2001/20/EC and the Helsinki Declaration for biomedical research involving humans. The University Ethics Committee approved the research protocol.

### The Testing Room and Instrumentation for the VOL Task

**FIGURE 1:**
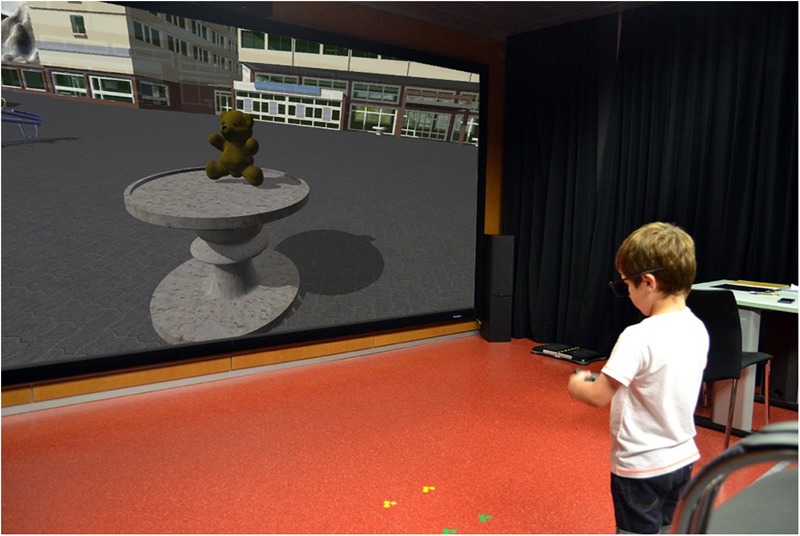
The testing room for the VOL task.

The testing room consisted of a square room of about 20 square meters (**Figure 1**). The child was placed in the middle of the room facing one of the walls on which was mounted a 120-inch screen. We used two projectors to send two images to the screen from the back. Each of the two projectors had a linear vertical polarizer. There was a difference of 90° between the directions of the two polarizers. The children wore linear polarized glasses to perceive the 3D sensation. These glasses had two vertical polarizers, one for each eye, that were aligned to match the directions of the projectors’ polarizers. The interface used to control the child’s movements in the VE was a PlayStation gamepad. We used Unity 3D as the game engine.

To run the application, we used a PC with an i7-4770k processor, 16 GB RAM memory, and a graphic card NVIDIA GTX770. The software and hardware used to develop the VOL task were described in Rodríguez-Andrés et al. (2016).

### The VOL Task

The task consisted of a short-term memory test for object location. Briefly, the participant had to search for objects that were placed on tables distributed in certain locations of a VE. Then, he/she had to remember their locations in order to place them in their correct positions later. There was a narrator who guided the participant through all phases of the task with her voice. She told the child what to do each time (e.g., “Remember the location of the objects that you are going to see now”; “Approach it and push the button when its color changes”; “You have to put this object in its correct position”). In Section “Training Phases and VOL Trials,” we briefly describe the phases of the VOL task. More details about the VOL task can be found in Rodríguez-Andrés et al. (2016).

#### Training Phases

**FIGURE 2:**
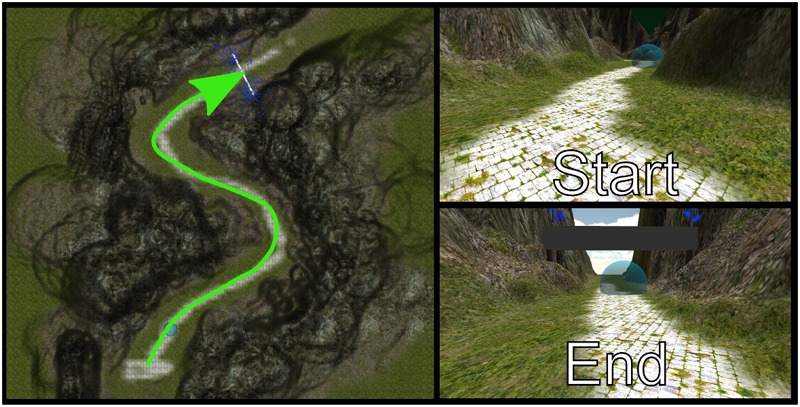
The VE in the mountains in the adaptation phase.

Before starting the trials of the VOL task, each child completed two separate phases: the adaptation phase and the tutorial phase. The aim of the adaptation phase was to familiarize the child with the interaction method. The child learned how to move inside a VE using the gamepad. In this phase, the participant was transported to a VE in mountainous terrain (**Figure 2**). Then, he/she had to follow a path across the mountains to arrive at a goal at the end of the path. Some arrows and bubbles showed the child which direction to follow.

In the tutorial phase, each child completed a short tutorial about how to perform the VOL task. He/she learned what the goal of the task was and how to achieve it. This phase was like a trial of the VOL task (see section “VOL Trials”) except for the fact that the child received a visual indication of the position of the object during the testing phase. The visual indication consisted of a vertical green arrow pointing to the position of the object.

#### VOL Trials

**FIGURE 3:**
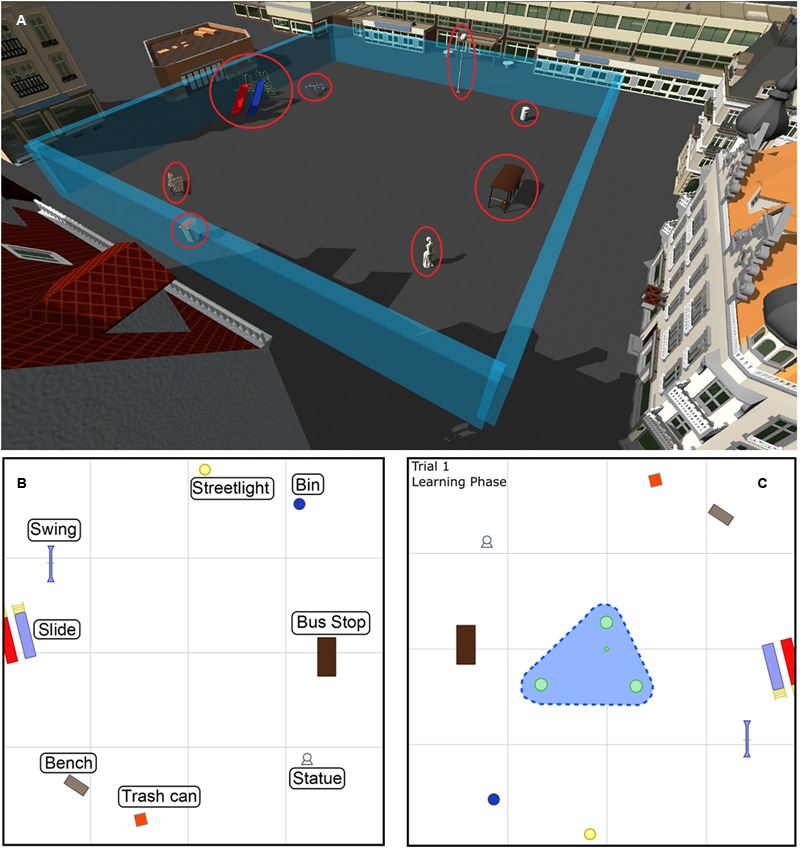
**(A)** The interaction area of the VOL task is delimited within imaginary blue walls, which are not visible to the participant. The objects that worked as proximal cues for orientation are indicated with red circles. **(B)** A schematic top view of the interaction area and the location of the proximal cues. **(C)** A schematic top view of the learning phase of Trial 1. An example showing the two separate areas: the peripheral area in white, and the central area in blue. The dashed blue line delimits the peripheral area.

The VE of the VOL task was simulated as a city square. The square was surrounded by several buildings (**Figure 3**). The child could move within the limits of an interaction area of the city square (**Figure 3A**). The buildings were outside of the interaction area of the child and worked as visual cues to help orient the participant with spatial orientation (distal cues). Inside the square, there were eight common objects of a city (proximal cues), which also served as guidance (**Figures 3A,B**). We defined two separate areas within the interaction area: the peripheral area, and the central area (**Figure 3C**). The peripheral area included a zone that was three meters away from the tables, whereas the central area included the area where objects were placed on tables (**Figure 3C**).

**FIGURE 4:**
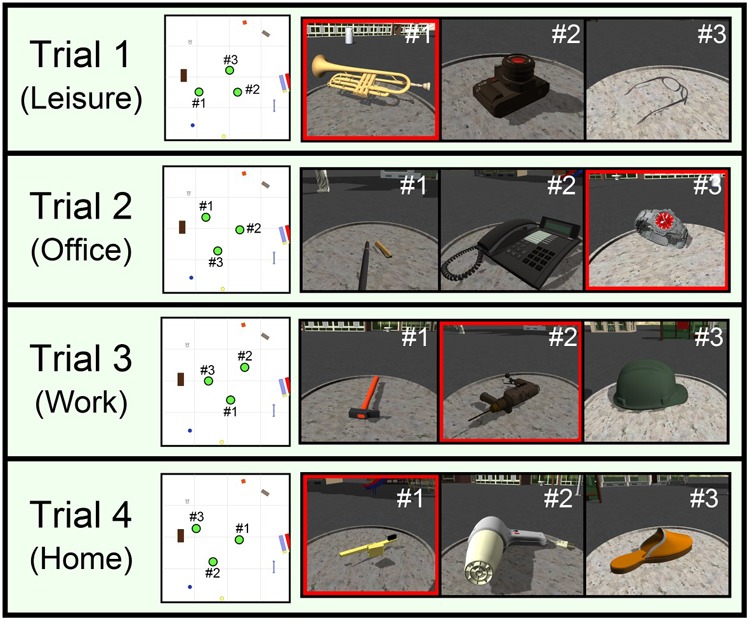
A general scheme of the four trials of the VOL task (Trials: 1–4), which shows the following information (from left to right): a schematic top view of the interaction area, and the location of the tables and the objects; an image of the objects numbered in order of appearance during the learning phase; and an image of the object asked about during the testing phase.

There were four VOL trials in the VOL task (see **Figure 4**). Each child completed these trials consecutively. The goal of these trials was to assess the children’s short-term memory for object location. Each trial was divided into two separate phases: the learning phase, and the testing phase.

Short-term memories for visuospatial items were formed in the learning phase. In this phase, the child searched three gray tables with the aim of finding three hidden objects. These objects were shown one by one. The system guided the search process using a green arrow that pointed to one of the gray tables. The child had to walk to the table, and, when the child was close enough, the table changed color from gray to green, and the child could see the object on that table for 5 s. The child had to repeat this process two more times to discover all of the objects. It is important to note that the children had to remember the objects they saw and where the objects were placed. At the end of each learning phase, the child returned to the center of the scene, and the VE was rotated 180° from the original position before starting the testing phase. Therefore, idiothetic information could not be used as a reference for orientation.

The testing phase consisted of the retrieval of the short-term memories for the visuospatial items that were formed in the learning phase. In this phase, the system showed an object on the screen and the narrator asked the participant for the position of that object. The participant had to put the object in its correct position to complete the trial. The position of the tables and the objects varied in the four different trials of the VOL task as shown in **Figure 4**. We included a score screen to keep the children motivated. They received a star when they finished the tutorial phase and each of the four VOL trials, regardless of the quality of their responses.

### Videogame Experience, 3D Perception, Satisfaction, and Usability Questionnaires

We designed a questionnaire to determine the participants’ videogame experience, consisting of two items: *“How often do you play with videogames on a PC or smartphone?”* and *“How often do you play videogames with a gamepad?”* The children answered the two items using a five-point Likert scale ranging from *“(1) Never”* to *“(5) Everyday.”* Also, the children gave their opinion about 3D perception during the performance on the VOL task by answering the statement *“At certain moments, the objects came out of the screen”* using a five-point Likert scale ranging from *“(1) Strongly disagree”* to *“(5) Strongly agree.”* The questionnaires were adapted to children. The items of the questionnaires were filled in using text labels that were accompanied by graphical icons (Read, 2008).

We collected information about the satisfaction and usability perceived by the children by using two questionnaires with five-point Likert scale items. The satisfaction questionnaire was made up of four items: *“How much fun did you have?”* [response scale: *“(1) None”* to *“(5) A lot”*], *“I would invite my friends to play the game”* [response scale: *“(1) Never”* to *“(5) Every day”*], *“Would you play this game another time?”* [response scale: *“(1) Never”* to *“(5) Every day”*], and *“Score the game from 1 to 5”* [response scale: *“(1) Very bad”* to *“(5) Very good”*]. Finally, a usability questionnaire had two items: *“Was the VOL task easy to play?”* [response scale: *“(1) Very difficult”* to *“(5) Very easy”*], and *“I always understood what I had to do”* [response scale: *“(1) Strongly disagree”* to *“(5) Strongly agree”*].

### Spatial Ability Tests

We used two classical psychometric tests to assess the children’s basic visuospatial abilities. We obtained an index of their visuospatial span with the forward version of the Corsi Block Tapping Test (CBTT), and we used the backward version of the CBTT to collect a measure of their visuospatial working memory capacity (Kessels et al., 2000). We also assessed their visuospatial and visuomotor ability with the Block Construction subtest (BC) from the Nepsy-II battery (Korkman et al., 2014).

### Emotional and Behavioral Rating Scale

We used the Parent Report form of the Behavioral Assessment Scale for Children (PRfBASC) (Reynolds and Kamphaus, 2004) to assess their emotional and behavioral outcomes. PRfBASC is one of the most widely used behavior rating scales for the assessment of behavioral problems, emotional symptoms, and adjustment patterns in children across the following domains of functioning: Internalizing, Externalizing, and Adaptive Behavior. The PRfBASC consists of 130 items (3–6 years old) or 134 items (6–12 years old) about a child’s behavior at home and in the community measured on a four-point Likert scale. In this study, we considered the following subscales of the PRfBASC: Anxiety, Depression, Hyperactivity, Aggressiveness, Withdrawal, and Attention Problems.

### Procedure

The participants were tested individually in sessions of approximately 55 min, which took place on the same day and between 9:00 A.M. and 2:00 P.M. They were randomly assigned to one of the following experimental conditions: Condition I and Condition II. In Condition I, the participants were tested with the Lang-Stereo-Test and then completed the questionnaire about videogame experience. Afterward, they performed the VOL task and then completed the questionnaires about 3D perception, satisfaction, and usability. Finally, they performed the CBTT and the BC. In Condition II, the participants performed the CBTT and the BC first and were then assessed with the remaining tests and questionnaires in the same order as described in Condition I. In the recruitment phase of the study (see section “Participants”), theparents completed the PRfBASC to obtain the emotional and behavioral measures. Before the child started, the child and his/her parents met the person responsible for the procedure, who accompanied the child during the whole session. The child and the experimenter talked for about 5 min, until the child felt comfortable with the situation. Then, the parents left the room and the session began.

### Data Analysis

We considered two variables that are related to the videogame experience questionnaire: (item 1) the child’s previous experience in playing videogames, and (item 2) child’s previous experience using the interaction method of the VOL task. We used the direct scores of these two items to calculate these variables. Similarly, we used the direct score of the 3D perception statement. For the satisfaction and usability questionnaires, we calculated the mean of the children’s direct scores for each item of these two questionnaires in order to obtain a general measure of satisfaction and usability in performing the VOL task.

We considered five variables that are related to the performance of the VOL task: VOL Task Score, Total Distance, Total Time Average Speed, and Peripheral Distance. The VOL Task score is an indicator of visuospatial memory and involved the sum of the trials of the VOL task that were correctly performed (range: 0–4). The Total Time consists of the time (in seconds) taken to complete the four trials of the VOL task. The Total Distance is the total distance (in virtual meters) traveled by the child in the four trials of the VOL task. The Average Speed is an indicator of the velocity (in virtual meters/sec) with which the child explored the VE. We calculated this variable by dividing the Total Distance traveled by the Total Time spent to perform the task. The Peripheral Distance consists of the distance traveled by the child in the peripheral zone of the interaction area in the four trials of the VOL task. For the variables: Total Distance, Total Time, Average Speed, and Peripheral Distance, we also calculated the values obtained by the sum of each phase of the VOL task separately (learning and testing).

For the measures of the visuospatial ability, we used the direct scores of the CBTT (forward and backward versions) and BC. Finally, the scores of the subscales measured with the PRfBASC-2 (Anxiety, Depression, Hyperactivity, Aggressiveness, Withdrawal, and Attention Problems) are reported as T-scores (*M* = 50, *SD* = 10).

We applied the Shapiro–Wilk test (Shapiro and Wilk, 1965) to check the normality distribution of the dataset variables. This test is especially powerful for samples of small size. The tests showed that only the Anxiety variable followed a normal distribution. We decided to perform non-parametric tests with the entire data-set which are more suitable with distributions of this kind. All analyses were done using the free Software R-Studio (Version 0.98.1079). The results were considered to be statistically significant if *p* < 0.05.

## Results

**FIGURE 5:**
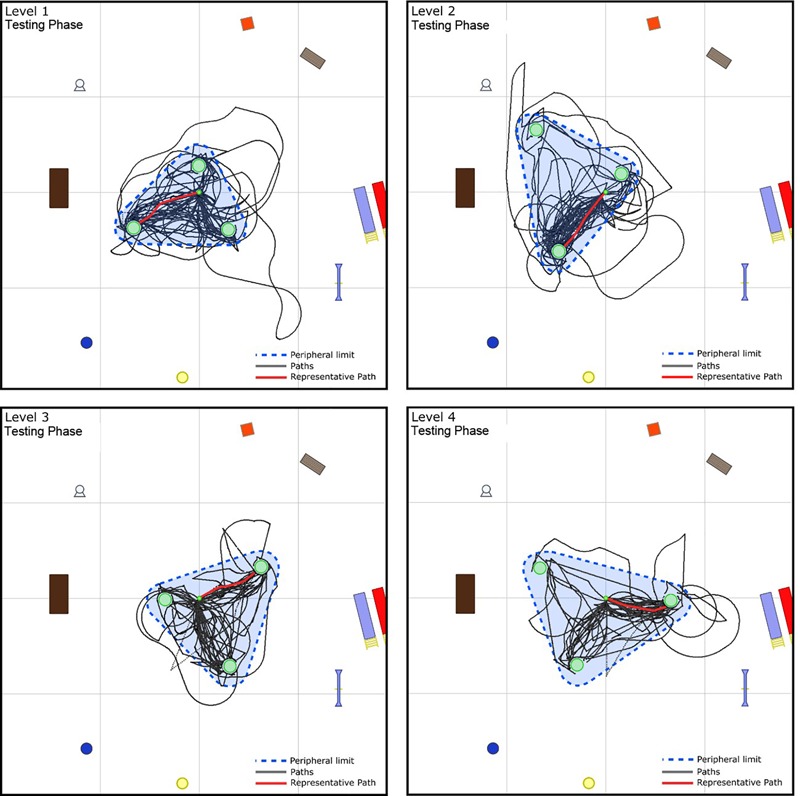
The paths made by the children in the testing phases of the four levels.

**Table 1** shows descriptive statistics for the five variables that are related to performance in the VOL task. In the case of time, speed, path length and peripheral path length, we present descriptive statistics for both the learning and testing phases of the VOL task. **Figure 5** shows the paths made by the children in the testing phases. **Table 1** also shows the descriptive statistics for the participants’ experience in playing videogames and using the interaction method, their 3D perception during the VOL task, and their perceived satisfaction and usability. **Table 2** shows the descriptive statistics for the children’s visuospatial abilities assessed with CBTT and CB, and their scores on the emotional and behavioral subscales of the PRfBASC-2.

**Table 1:** Mean scores (standard deviations) for the variables of the VOL task, videogame experience, 3D perception, satisfaction, and usability questionnaires (*N* = 52).

Type of measure (range/unit)	*M (SD)*
Performance on the VOL task	
VOL Task Score (0–4)	2.63 (1.23)
Total Time (seconds)	498.12 (219.67)
Total Time – learning phase (seconds)	337.80 (132.54)
Total Time – testing phase (seconds)	120.32 (105.87)
Average Speed (meters/second)	5.164 (1.921)
Average Speed – learning phase (meters/second)	4.666 (1.396)
Average Speed – testing phase (meters/second)	6.134 (3.438)
Total Distance (meters)	2352.7 (747.5)
Total Distance – learning phase (meters)	1598.7 (452.0)
Total Distance – testing phase (meters)	520.2 (256.0)
Peripheral Distance (meters)	712.4 (753.3)
Peripheral Distance – learning phase (meters)	197.2 (254.8)
Peripheral Distance – testing phase (meters)	53.7 (99.4)
Videogame experience	
Experience in Videogames (1–5)	3.44 (0.93)
Interaction Method Experience (1–5)	2.08 (1.00)
Perception about VOL task	
3D perception (1–5)	3.56 (1.41)
Satisfaction (1–5)	3.39 (1.09)
Usability (1–5)	4.44 (0.48)

**Table 2:** Mean scores (standard deviations) for the spatial ability tests and the subscales of the Parent Report form of the BASC-2 used in the study (*N* = 52).

Type of measure	Test/subscale	*M (SD)*
Visuospatial abilities		
	CBTT forward	5.21 (1.01)
	CBTT backward	4.62 (1.00)
	BC	14.75 (4.46)
Emotional and behavioral outcomes		
	PRfBASC-2 subscales:	
	Anxiety	44.25 (8.88)
	Depression	47.77 (8.07)
	Hyperactivity	46.77 (8.08)
	Aggressiveness	49.33 (9.00)
	Withdrawal	48.48 (9.22)
	Attention Problems	48.65 (8.22)

*CBTT, Corsi Block Tapping Test; BC, Block Construction subtest from the Nepsy-II; PRfBASC, Parent Report form of the Behavioral Assessment Scale for Children.*

### Effects of Age, Gender, and Previous Videogame Experience on Performance in the VOL Task

**FIGURE 6:**
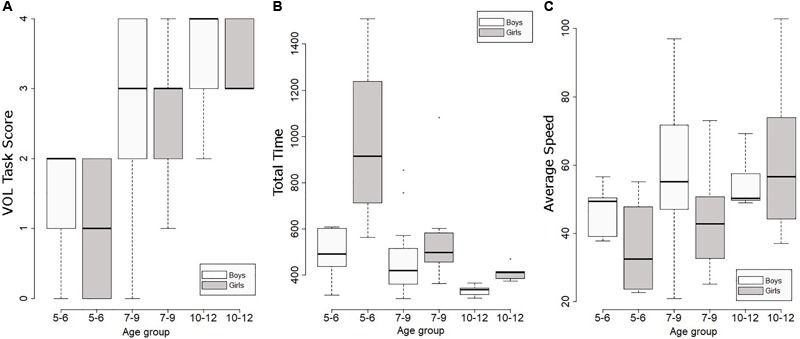
**(A)** Boxplots of the VOL Task Score; **(B)** Total Time; and **(C)** Average Speed of the three age groups separated by gender.

The task performance variables were analyzed using the Kruskal–Wallis test with four factors: Gender, Age, Experience in Videogames, and Interaction Method Experience. **Table 3** shows the results of the statistical analyses. The Kruskal–Wallis test revealed a significant effect of Age group on the VOL Task Score. The older children had higher scores than the younger ones [χ^2^(2) = 15.8, *p* < 0.01]. A *post hoc* test showed significant differences between Age 5–6 and 7–9 (*r* = 0.49, *p* < 0.001), and between Age 5–6 and 10–12 (*r* = 0.84, *p* < 0.001). The test also indicated that the younger children spent more time completing the task [χ^2^(2) = 13.98, *p* < 0.01]. **Figure 6A** shows the influence of Gender and Age on the VOL Task Score. The gray and white boxes of the same age group are placed at the same height. The boxes are closer to the maximum score in the oldest group.

**Table 3:** The results of the Kruskal–Wallis tests for the variables related to the performance of the VOL task.

Variable	Factor	χ^2^	*df*	*p*-value
VOL Task Score				
	Age Group	**15.79**	**2**	**<0.001**
	Gender	0.33	1	0.57
	Experience in Videogames	3.37	4	0.50
	Interaction Method Experience	7.85	3	0.05
Total Distance				
	Age Group	3.45	2	0.18
	Gender	0.73	1	0.39
	Experience in Videogames	0.96	4	0.91
	Interaction Method Experience	1.64	3	0.65
Total Time				
	Age Group	**13.98**	**2**	**<0.001**
	Gender	**7.11**	**1**	**0.007**
	Experience in Videogames	7.87	4	0.10
	Interaction Method Experience	4.54	3	0.21
Average Speed				
	Age Group	3.67	2	0.16
	Gender	**4.08**	**1**	**0.04**
	Experience in Videogames	**12.25**	**4**	**<0.01**
	Interaction Method Experience	6.34	3	0.10
Peripheral Distance				
	Age Group	2.88	2	0.24
	Gender	0.66	1	0.41
	Experience in Videogames	1.44	4	0.84
	Interaction Method Experience	0.65	3	0.88

*The tests that reached significance are displayed in bold type.*

The Kruskal–Wallis tests also revealed that there was a significant effect of Age on the Total time spent to complete the task. The younger children required more time than the older ones [χ^2^(2) = 13.98, *p* < 0.01]. There are statistically significant differences related to the Gender factor. The girls spent more time than the boys to complete the task in all age groups (**Figure 6B**). This difference was especially high in the 5 to 6 year-old group. The girls in this group spent a mean of 16 min to complete the entire task.

To check if previous experience in videogames or previous experience with the interaction method influenced performance on the VOL task, we included these variables in the analyses. The Kruskal–Wallis tests show that only the average speed in the task is influenced by previous experience with videogames [χ^2^(4) = 12.25, *p* < 0.01]; the children who had more experience with videogames completed the task faster than those who did not have as much experience. Previous experience with the interaction method did not influence any of the variables considered (*p* > 0.05).

### Usability, Satisfaction, and 3D Perception

We performed one Kruskal–Wallis test for each VOL task measure of performance, using Usability, Satisfaction, and 3D perception as dependent variables. The results of these tests are shown in **Table 4**. The tests indicated that there were no statistically significant differences in the measures of the VOL task performance in relation to these variables. These results reflect that the users’ perception of the task and the system did not influence the way users performed the task.

**Table 4:** Multifactorial Kruskal–Wallis tests for the Usability, Satisfaction, and 3D Perception variables.

Variable	Factor	χ^2^	*df*	*p*-value	Significance
Usability					
	VOL Task Score	0.05	3	0.99	–
	Total Distance	3.30	3	0.65	–
	Total Time	0.16	3	0.98	–
	Average Speed	2.95	3	0.40	–
	Peripheral Distance	2.75	3	0.43	–
Satisfaction					
	VOL Task Score	3.13	13	0.99	–
	Total Distance	10.41	13	0.66	–
	Total Time	9.97	13	0.70	–
	Average Speed	19.63	13	0.10	–
	Peripheral Distance	13.64	13	0.40	–
3D Perception					
	VOL Task Score	7.02	4	0.13	–
	Total Distance	20.01	4	0.73	–
	Total Time	5.89	4	0.21	–
	Average Speed	1.84	4	0.76	–
	Peripheral Distance	3.61	4	0.46	–

### Relationship Between Performance on the VOL Task and the Participant’s Outcomes

To determine the relations between the different performance outcomes in the VOL task and the different scores obtained depending on videogame experience, perception of the VOL task, visuospatial ability, and emotional and behavioral variables, we performed a partial Spearman correlation extracting the influence of Age (**Table 5**).

**Table 5:** Partial Spearman correlations (*N* = 52).

VOL task variables		VOL score	Total time	Average speed	Total distance	Peripheral distance
**Videogame experience variables:**
Experience in Videogames	*r (p)*	0.06 (0.06)	0.05 (0.72)	-0.07 (0.63)	0.00 (0.99)	0.04 (0.78)
Interaction Method Experience	*r (p)*	0.08 (0.67)	-**0.29 (0.04)**	**0.30 (0.03)**	0.04 (0.76)	0.08 (0.57)
**Perception about VOL task:**
3D Perception	*r (p)*	-0.13 (0.37)	0.02 (0.89)	-0.07 (0.63)	-0.14 (0.33)	0.05 (0.71)
Satisfaction	*r (p)*	-0.07 (0.59)	0.02 (0.90)	0.00 (0.97)	-0.01 (0.93)	-0.04 (0.78)
Usability	*r (p)*	0.00 (0.94)	-0.15 (0.29)	-0.04 (0.79)	-0.25 (0.07)	-0.21 (0.14)
**Visuospatial ability variables:**
CBTT forward score	*r (p)*	0.19 (0.18)	-0.14 (0.31)	-0.18 (0.22)	-**0.28 (0.04)**	-**0.31 (0.03)**
CBTT backward score	*r (p)*	**0.29 (0.04)**	0.03 (0.86)	0.14 (0.33)	0.08 (0.57)	0.05 (0.72)
BC score	*r (p)*	**0.43 (< 0.01)**	0.01 (1.00)	0.10 (0.50)	-0.02 (0.87)	0.05 (0.71)
**Emotional and behavioral variables:**
Anxiety	*r (p)*	-0.10 (0.47)	-0.18 (0.19)	-0.01 (0.92)	-0.02 (0.87)	0.05 (0.71)
Depression	*r (p)*	-0.08 (0.56)	0.04 (0.79)	0.03 (0.82)	0.23 (0.10)	0.11 (0.44)
Hyperactivity	*r (p)*	-0.05 (0.71)	-0.12 (0.41)	0.09 (0.52)	-0.02 (0.87)	-0.09 (0.51)
Aggressiveness	*r (p)*	0.03 (0.83)	-0.18 (0.21)	**0.30 (0.04)**	0.21 (0.13)	0.10 (0.49)
Withdrawal	*r (p)*	-0.01 (0.96)	0.11 (0.43)	0.11 (0.44)	**0.31 (0.03)**	**0.31 (0.03)**
Attention Problems	*r (p)*	-0.17 (0.23)	0.00 (0.98)	0.20 (0.15)	**0.32 (0.02)**	0.10 (0.49)

*CBTT, Corsi Block Tapping Test; BC, Block Construction subtest from the Nepsy-II. The correlation coefficients that reached significance are displayed in bold type.*

Some displacement indicators in the VE showed significant correlations. There were significant direct correlations between the VOL task score and two variables of visuospatial abilities in small-scale real space: the visuospatial span backward (CBTT backward; Spearman’s *r* = 0.29, *p* = 0.04), and blocks construction (BC; Spearman’s *r* = 0.43, *p* < 0.01). Furthermore, there were relations between the Total Distance (Spearman’s *r* = -0.27, *p* = 0.04) and Peripheral Distance (Spearman’s *r* = -0.31, *p* = 0.03) with the CBTT forward score. We also found that with for those with less experience with gamepads, the completion time for the VOL task was longer (Spearman’s *r* = -0.29, *p* = 0.04).

There were significant direct correlations between the Average Speed of navigation in the VE and experience with the gamepad interaction (Spearman’s *r* = 0.30, *p* = 0.03). The same type of meaningful relationship was found between Average Speed and the score on the Aggressiveness subscale (Spearman’s *r* = 0.30, *p* = 0.04). Longer navigation paths were related to higher scores on the Withdrawal (Spearman’s *r* = 0.31, *p* = 0.03) and Attention Problem subscales (Spearman’s *r* = 0.32, *p* = 0.02). In addition, longer navigation paths in the peripheral area of the VE were related to higher scores on the Withdrawal subscale (Spearman’s *r* = 0.31, *p* = 0.03). Shorter path lengths in both the whole interaction area and in the peripheral area of the VE were related to higher visuospatial span scores measured with the CBTT forward (Spearman’s *r* = -0.31, *p* = 0.03).

## Discussion

We studied the performance of typically-developing boys and girls in a VE that was designed to test short-term memory for the location of objects placed in specific places in a city square (i.e., the VOL task). The VE worked as an open field, which included proximal and distal cues that are common in a city. The area of interaction was divided into two areas (i.e., central and peripheral). The VE was actively explored using a gamepad. We considered participants’ age, gender, and previous videogame experience as potential variables that could influence success on the VOL task and the way of exploring the VE. We also examined relationships among the variables in performance on the VOL task and visuospatial, emotional, and behavioral outcomes.

The children’s performance on the VOL task and their visuospatial skills correlated. The task also obtained high values of usability and satisfaction by the children. Hence, we considered that the task was appropriate for studying the spatial performance in a child population without disabilities.

As we hypothesized, the participants’ age affected their performance on the VOL task. The task involved the retrieval of short-term memories of three visuospatial items. Also, the proximal and distal cues were important for orientation. The children could not use a strategy based on routes since their point-of-view position was rotated between the learning and testing phases. Their success was dependent on the creation and use of a mental map of the city square and/or links between the target and its surrounding cues. The lower scores of the youngest participants suggest that their visuospatial short-term memory and/or their spatial strategies were relatively weaker during this developmental period. This result is consistent with previous studies (Bullens et al., 2010; Purser et al., 2012; Nys et al., 2015; Mendez-Lopez et al., 2016; Merrill et al., 2016) and with the results found by Rodríguez-Andrés et al. (2016). They performed descriptive analyses taking into account the age of the participants and the VOL task score. They found a trend toward a better score on the task by the older children than the younger ones.

Age and gender also affected the total time spent on the task. This time was especially longer in the youngest group studied and was related to gender differences found in navigation speed (**Figure 6C**) and amount of previous experience with videogames. Children who played videogames frequently were found to navigate with greater speed, precision, and agility. The amount of experience with technological devices increases with the age (Sayers, 2004). Also, girls play videogames less frequently (Terlecki and Newcombe, 2005). Interestingly, experience with playing videogames influenced only performance variables that reflected the way in which the participant explored the VE but did not affect the score obtained. The VOL score was a measure of the visuospatial abilities of the children based on the correlations found between this outcome and the score obtained on the paper–pencil spatial tests. This result suggests that being less skilled in videogames does not influence the visuospatial ability of the user obtained in a virtual spatial task, but it does influence the speed of exploration of the VE.

Familiarity with the interaction method used in the VOL task did not impair how the way the children explored the space; however, there was a trend toward a lower ability to locate the objects by the less experienced participants. Also, those more skilled with the gamepad were faster in the completion of the task and the navigation of the VE, but the strength of the correlation was weak. Our sample was not very familiar with the gamepad overall. The gamepad was the preferred device of interaction by children from 5 to 10 years old when compared with a device based on a natural user interface in a previous study (Rodríguez-Andrés et al., 2016). As noted by the mean score, our participants had played with a gamepad occasionally, but their frequency of use was less than once a week. Based on our informal notes, they played more frequently using touch-based interfaces in mobile devices, including tablets. We gave them training to reduce the possibility of differences in experience-based performance (Sandamas et al., 2009). The VOL task included two phases for practice with the interface (i.e., adaptation and tutorial). The first one involved practicing in a VE which was more difficult to explore than the VE of the learning trials. The VE of the adaptation phase required strong fine motor skills. These two phases gave the users training in the procedural aspects of the virtual navigation. In this way, we attempted to reduce any potential bias due to experiential factors in the interpretation of children’s ability to locate objects.

The children’s perception of the task experience and with overall system was very positive, especially for its usability aspects. The children gave a score close to maximum on the usability questionnaire; he means were 4 on a scale from 1 to 5. This result shows that the task was easy for them to perform. Three reasons for the high usability level include (1) the procedure aimed to facilitate the familiarity and comprehension of task phases, (2) the users were habituated to the interaction system prior to being tested, and (3) the children appreciated the innovative nature of the system. For example, the 120-inch stereoscopic screen, wearing polarized glasses, and the sense of immersion in the virtual city were novelties for most if not all of the children (Wells et al., 2010). However, we suggest that novelty alone is unlikely to influence the positive scores. If that were the case, the children would have given the maximum score on the satisfaction and 3D perception questionnaires. All of the children had stereoptic vision, but their 3D perception was not highly positive considering their mean rating of the experience (3.56 out of a maximum of 5). Similarly, the children were satisfied with the task, but some aspects of the task might have increased the perceived satisfaction more than others. The task provided motivating feedback after the completion of each trial regardless of the quality of execution. This was to prevent any frustration that might have been caused by a feeling of inadequacy and to keep the children engaged throughout the task. Despite this, the children gave the task a relatively high score (3.39 out of 5).

As we mentioned above, boys were more skilled than girls in the exploring the VE. However, contrary to what we expected, boys did not outperform girls in their ability to locate the virtual objects. The similar performance between boys and girls was also found in several studies in which children were trained in a navigational short-term memory task (Juan et al., 2014; León et al., 2014; Piccardi et al., 2014; Mendez-Lopez et al., 2016). Also, Rodríguez-Andrés et al. (2016) did not find significant differences between 5 and 10-year old boys and girls in their ability to locate the objects in the task. They performed similarly regardless of the type of interaction used (i.e., natural interaction or gamepad). The level of difficulty of the VOL task was low in terms of its VE and the memory load required. The spatial layout of the VE had proximal and distal cues guiding orientation. All of these cues could be seen from any viewpoint of the interaction area by the rotation of the VE during the exploration. In addition, the task requirement was to store three spatial locations temporarily that had been sequentially presented. The results agree with those of León et al. (2014) who suggested that gender differences emerge only in spatial tasks that are more challenging.

We found significant correlations between task performance and children’s visuospatial abilities in some paper and pencil spatial tests. The moderate strength of the correlation found between the VOL score and the score on the BC subtest indicates that the success in object location in our task is related to the general ability to calculate position and directionality (Korkman et al., 2014). It is also related to spatial working memory, but to a lesser extent as indicated by the weak correlation found between the VOL score and the score on the CBTT backward subtest. High scores on this subtest reveal good skill in holding in mind and manipulating a large number of visuospatial items (Kessels et al., 2000). We suggest that the mental manipulation of spatial representations is a key factor in solving our virtual task because there was no correlation with the simple ability to maintain spatial information in short-term memory. This supports the relevance of executive functions in working memory tasks involving spatial relations (Purser et al., 2012; Korthauer et al., 2017). It is also interesting to note that those participants with lower spatial spans executed longer path lengths in the VE, but the strength of the correlation was weak. The spatial span measure is affected by attentional capacity (Kessels et al., 2000), thus the result could also be interpreted as reflecting the relation between execution of longer paths and increased attentional difficulties. Those children that are more prone to distraction might be less able to navigate efficiently to a spatial target. Thus, the level of the participants’ attentional capacity could be an important factor to be considered in spatial task performance, as discussed by Farran et al. (2015).

Contrary to what we expected, we did not find correlations between anxiety measures and displacements within the peripheral area of the VE. We propose three possible explanations for this result: (1) the children scored within the normal limits in all emotional and behavioral outcomes considered, whereas previous studies were conducted using samples with clinical symptoms (Mueller et al., 2009; Burles et al., 2014), (2) our VE was emotionally neutral, and we gave children the optimal conditions to reduce anxiety regarding the testing situation (i.e., an initial familiarization with the experimenter and positive reinforcement during the task), and (3) our VE target items were all in the same space. A more complex environment with several spaces that are not accessible at first sight might be more prone to reveal significant correlations with anxious behavioral traits.

Nevertheless, withdrawal behaviors were related to an increase in the exploratory behaviors in our sample, but they did not affect spatial learning or the time spent on the task. It might be suggested that the children withdraw made an intense exploration of the VE in order to achieve a good spatial representation. Fornasari et al. (2013) found an effect of withdrawal on the exploration of a virtual city. Contrary to what we found, this behavior was related to a reduction in the exploration of the VE, but the differences between these results could be explained by the populations studied. In the case of Fornasari et al. (2013), they studied a clinical population of children with autism. Based on levels of withdrawal within the normal limits, we can speculate that withdrawal might have a negative impact on social outdoor games. This result partially supported the relevance of previous experiences in spatial behaviors proposed by other authors (e.g., Lawton and Kallai, 2002).

Finally, those children who are more prone to aggressive behaviors navigated the VE faster, but there was no significant correlation with time savings. This result is in line with studies confirming that the feeling of anger predicts faster motor behavior (e.g., Deffenbacher et al., 2003; Roidl et al., 2014).

The present research has some limitations. First, our task tests spatial short-term memory learning for three locations in a VE that works like an open field. The level of difficulty is low. It would have been interesting to compare results with those obtained in a task that was more difficult and a VE that was more complex. The second problem is related to the sample. It would have been desirable to increase the number of participants in each age group, especially in the youngest group.

The VOL task presents a VE in which participants use their navigational competencies and their spatial short-term memory for the location of objects. The key factor in an object-location task is the possibility to mentally represent a spatial configuration of interrelated objects. We used three objects because previous research has tested adults in spatial tasks with three or four objects (Zimmer et al., 2003; Iachini et al., 2009), and, from 5 years of age, a person is able to recall the location of 2–3 objects (Mendez-Lopez et al., 2016). The VOL task is attractive for children and is also challenging for adults. We considered that this is a positive aspect of this task because the VOL task provides an opportunity to increase knowledge about spatial memory and navigation and to directly compare these skills in participants of all ages. However, this aspect also puts us at a disadvantage in determining the effects of individual factors in spatial performance. If the task had incorporated more objects that would make it more difficult to perform, there might have been gender effects in favor of the boys. As we have discussed above, the difficulty of a spatial task is a key factor in the existence of gender differences (León et al., 2014). Also, we hypothesize that an increase in the number of objects to recall would have negatively affected the scores on satisfaction and usability given by the younger children because it would be very difficult for them to perform it. In addition, their exploratory behavior would have been more prone to reveal significant correlations with anxiety or withdrawal behaviors.

## Conclusion

Age affected the spatial short-term memory for the location of three objects in a virtual city in children between the ages of 5 and 12 years. Three factors contributed positively to improving the accuracy of the children’s performance during the navigation: age, being male, and having more experience with videogames. There were weak associations among variables which showed that the individual differences in visuospatial skills correlated mainly with the ability to recall objects’ positions. Behavioral and emotional variables were not related to object location memory. However, three variables were associated with differences in the exploration of the VE, namely: levels of attention, aggressiveness, and withdrawal.

## Author Contributions

DR-A, MM-L, M-CJ, and EP-H: conceived, designed, and performed the experiments, interpreted the data, and drafted the manuscript. DR-A and MM-L: analyzed the data.

## Conflict of Interest Statement

The authors declare that the research was conducted in the absence of any commercial or financial relationships that could be construed as a potential conflict of interest.
